# Antidiabetic Effects of Yam (*Dioscorea batatas*) and Its Active Constituent, Allantoin, in a Rat Model of Streptozotocin-Induced Diabetes

**DOI:** 10.3390/nu7105411

**Published:** 2015-10-15

**Authors:** Hyeon-Kyu Go, Md. Mahbubur Rahman, Gi-Beum Kim, Chong-Sam Na, Choon-Ho Song, Jin-Shang Kim, Shang-Jin Kim, Hyung-Sub Kang

**Affiliations:** 1 Department of Veterinary Pharmacology and Toxicology, College of Veterinary Medicine, Chonbuk National University, Iksan Campus, 79 Gobong-ro, Iksan-si, Jeollabuk-do 570-752, Korea; govet@kamsi.co.kr (H.-K.G.); mahbubs84@gmail.com (M.M.R.); kgb70@jbnu.ac.kr (G.-B.K.); kimjs@chonbuk.ac.kr (J.-S.K.); 2 Department of Animal Biotechnology, College of Agriculture and Life Science, Chonbuk National University, 567 Baekje-daero, Deokjin-gu, Jeonju-si, Jeollabuk-do 561-756, Korea; csna@jbnu.ac.kr; 3 Department of Food Resource Marketing Economics, College of Environmental and Bioresource Sciences, Chonbuk National University, 567 Baekje-daero, Deokjin-gu, Jeonju-si, Jeollabuk-do 561-756, Korea; mento@jbnu.ac.kr

**Keywords:** *Dioscorea batatas*, diabetic, insulin, hyperglycemia, glucagon-like peptide-1, antioxidant, rat

## Abstract

The objective of this study was to investigate the therapeutic efficacies of crude yam (*Dioscorea batatas*) powder (PY), water extract of yam (EY), and allantoin (the active constituent of yam) in streptozotocin (STZ)-induced diabetic rats with respect to glucose, insulin, glucagon-like peptide-1 (GLP-1), C-peptide, glycated hemoglobin (HbAlc), lipid metabolism, and oxidative stress. For this purpose, 50 rats were divided into five groups: normal control (NC), diabetic control (STZ), and STZ plus treatment groups (STZ + PY, STZ + EY, and STZ + allantoin). After treatment for one-month, there was a decrease in blood glucose: 385 ± 7 in STZ, 231 ± 3 in STZ + PY, 214 ± 11 in STZ + EY, and 243 ± 6 mg/dL in STZ + allantoin, respectively. There were significant statistical differences (*p* < 0.001) compared to STZ (100%): 60% in STZ + PY, 55% in STZ + EY, and 63% in STZ + allantoin. With groups in the same order, there were significant decreases (*p* < 0.001) in HbAlc (100% as 24.4 ± 0.6 ng/mL, 78%, 75%, and 77%), total cholesterol (100% as 122 ± 3 mg/dL, 70%, 67%, and 69%), and low-density lipoprotein (100% as 29 ± 1 mg/dL, 45%, 48%, and 38%). There were also significant increases (*p* < 0.001) in insulin (100% as 0.22 ± 0.00 ng/mL, 173%, 209%, and 177%), GLP-1 (100% as 18.4 ± 0.7 pmol/mL, 160%, 166%, and 162%), and C-peptide (100% as 2.56 ± 0.10 ng/mL, 129%, 132%, and 130%). The treatment effectively ameliorated antioxidant stress as shown by a significant decrease (*p* < 0.001) in malondialdehyde (100% as 7.25 ± 0.11 nmol/mL, 87%, 86%, and 85%) together with increases (*p* < 0.01) in superoxide dismutase (100% as 167 ± 6 IU/mL, 147%, 159%, and 145%) and reduced glutathione (100% as 167 ± 6 nmol/mL, 123%, 141%, and 140%). The results indicate that yam and allantoin have antidiabetic effects by modulating antioxidant activities, lipid profiles and by promoting the release of GLP-1, thereby improving the function of β-cells maintaining normal insulin and glucose levels.

## 1. Introduction

Diabetes is a chronic endocrine disease characterized by persistent hyperglycemia and associated with abnormalities of carbohydrate, protein, and lipid metabolism. This disease is caused by a decrease or deficiency of insulin secretion and/or from increased cellular resistance [1]. Diabetes poses a serious threat to health worldwide due to its severe effects on the micro- and macrovascular systems. Specifically, diabetes can cause damage and/or dysfunction of multiple organs and tissues, especially the eyes, kidneys, nerves, heart, and blood vessels [1]. The total number of people with diabetes is projected to rise from 171 million in 2000 to 366 million in 2030 [2]. Controlling blood glucose levels is essential for preventing diabetic complications and for improving the health of patients with diabetes. Currently available drugs for diabetes, however, have a number of limitations, such as unwanted side effects that include hypoglycemia, cell death, and high rates of secondary failure [3]. Recent efforts for the complementary treatment of diabetes have focused on functional foods and their bioactive compounds [4].

Yam, *Dioscorea batatas* (*D. batatas*), belongs to the *Dioscoreaceae* family and has been widely used to promote health and in Asian traditional medicine for the treatment of several illnesses such as hypertension, asthma, abscesses, chronic diarrhea, diabetes, inflammation, cancer, and ulcers [5,6,7,8]. The major constituents of yam are allantoin, dioscorin, sapogenins, prosapogenin, gracillin, choline, l-arginine, polysaccharides, and proteins [4,8,9,10,11]. Due to their immunomodulatory and antitumor effects, yam polysaccharides have attracted increasing attention in the biochemical and medical fields [9]. Yam mucopolysaccharide has been demonstrated to possess immunostimulating bioactivities, including enhancing INF-γ secretion by splenic lymphocytes, stimulating phagocytosis, and aiding macrophages [9]. Choline is known to regulate the functioning of the peripheral and central nervous systems. l-arginine has been shown to boost the immune system and to be useful in treating cancer [10]. Allantoin is nature-identical, safe, and nontoxic; moreover, this substance is effective for treating diabetes, hypertension, cancer, and common aches [6,7,10].

Previous studies have demonstrated that the ability of yam to lower blood glucose is related to improved glucose metabolism via the up-regulation of the plasma membrane receptor GLUT4 [4,12]. Yam also improves intestinal Na^+^/K^+^ ATPase activity [8] by activating imidazoline I-2 receptors [13]. However, the mechanisms by which yam improve the biochemical profiles of patients with diabetes have not yet been completely characterized. Decreased levels of insulin, glucagon-like peptide-1 (GLP-1), and C-peptide; increased levels of glycated hemoglobin (HbAlc) and oxidative stress markers; and imbalanced lipid profiles, electrolyte levels, and antioxidant activities are associated with diabetes. Based on these observations, the objective of this study was to investigate the therapeutic efficacies of crude yam (*Dioscorea batatas*) powder (PY), water extract of yam (EY), and allantoin (the active constituent of yam) in streptozotocin-induced diabetic rats with respect to GLP-1, C-peptide, HbAlc, oxidative stress, insulin, glucose, and lipid metabolism

## 2. Experimental Section

### 2.1. Plant Materials and Preparation of Water Extract

Powdered crude yam (*Dioscorea batatas*) was obtained from the Seodongma Local Industry Project Association, Inc. (Iksan, Republic of Korea). A total amount of 10 g of crude yam powder (PY) was suspended in 1000 mL of distilled water, heated at 80 °C, stirred continuously for 48 h, and filtered. The resulting filtrate was sequentially concentrated in a vacuum rotary evaporator and further freeze-dried to obtain the water extract of yam (EY, 4.73 g). This extract was freshly dissolved in distilled water before administration.

### 2.2. Experimental Animals

Male Sprague-Dawley rats weighing 200–250 g were purchased from Koatech (Gyeonggi-do, Korea). Rats were in housed controlled humidity (60%–70%) and temperature (23 ± 2 °C) conditions with a 12 h light/dark cycle. Rats had free access to a standard rat pellet diet and tap water. All experimental protocols (CBU2013-0010) were approved by the Committee on the Care of Laboratory Animal Resources, Chonbuk National University and were conducted in accordance with the Guide for the Care and Use of Laboratory Animals [14].

### 2.3. Induction of Experimental Diabetes Mellitus and Experimental Design

In total, 50 rats were divided equally into five groups: normal control, streptozotocin (STZ)-induced diabetic control, suspended crude yam powder-treated diabetic (STZ + PY), water extract of yam-treated diabetic (STZ + EY), and allantoin-treated diabetic group (STZ + Allantoin). Diabetes was induced by intraperitoneal injection of STZ (Sigma-Aldrich, St. Louis, MO, USA) at a dose of 50 mg/kg body weight (BW). For this purpose, STZ was dissolved in 0.1 mol/L sodium citrate buffer (pH 4.5). Three days after the induction of diabetes, rats with blood glucose levels over 350 mg/dL were considered to have type 1-like diabetes mellitus. Once diabetes was established, distilled water (DW) and yam were administered by gavage needle (*Per Os*) for 31 days. The daily doses of PY, EY, and allantoin were 1000 mg/kg BW, 500 mg/kg BW, and 2 mg/kg BW, respectively. PY and EY were orally administrated, whereas allantoin (Sigma-Aldrich, St. Louis, MO, USA) was injected intraperitoneally because it degrades in the GI tract [4]. The NC group treated only with equal volume of DW. Yam (*Dioscorea* spp.) has been reported to contain a higher allantoin content than any other plant; moreover, allantoin has been identified as an abundant and active constituent of yam. A previous study reported that the amount of allantoin in *D. batatas* ranged from 4.1 to 7.1 mg/g dry weight [15]. In a previous study, 0.5 mg/kgBW allantoin was administered intravenously to STZ-induced diabetic rats [4]. Due to the high amount of moisture (about 80%) in *D. batatas* [16], in this study 2 mg/kg BW of allantoin and 1000 mg/kg BW of PY were used. Since the yield of EY was about 50% that of PY, 500 mg/kg BW was selected as the daily dose of EY. All animals were carefully monitored on a daily basis for the duration of the study.

### 2.4. Measurement of Blood Glucose Levels

Fresh blood samples were collected from the tail vein of the rats after 12 h fast. The fasting blood glucose (FBG) levels were determined with a blood glucose meter (ACCU-CHEK^®^ Active, Roche Diagnostics, Mannheim, Germany). These measurements were performed on days 0, 11, 21, and 31.

### 2.5. Biochemical Analysis

Blood was collected from the caudal vena cava after anesthesia with a mixture of Zoletil and Rompun (30 and 5 mg/kg BW, respectively). Blood collection, storage, and measurement were performed as previously described [16]. A Nova Stat Profile^®^ pHOx^®^ Ultra analyzer (NOVA Biomedical Corp., Waltham, MA, USA) was used to measure the levels of lactate, pH, HCO_3_^−^, hemoglobin, hematocrit, ionized magnesium (Mg^2+^), calcium (Ca^2+^), potassium (K^+^), and chloride (Cl^−^) in freshly collected whole blood. As previously described [17], the anion gap values were calculated by the formula, (Na^+^ − (Cl^−^ + HCO_3_^−^)).

After clotting, blood serum was separated by centrifugation at 3000 rpm for 20 min. The levels of alanine aminotransferase (ALT), aspartate aminotransferase (AST), alkaline phosphatase (ALP), lactate dehydrogenase (LDH), creatinine kinase (CK), albumin, total cholesterol (TC), total protein (TP), triglyceride (TG), high-density lipoprotein (HDL), low density lipoprotein (LDL), creatinine (CRE), blood urea nitrogen (BUN), and uric acid (UA) were analyzed using a Model 7020 auto analyzer (Hitachi, Tokyo, Japan). Osmolality (Osm) values were calculated by the formula, (1.86 × Na^+^ + (Glucose/18) + (BUN/2.8) + 9) as previously described [17].

### 2.6. Serum Insulin, Nitrite/Nitrate, GLP-1, C-Peptide, and HbAlc Measurements

Fasting serum insulin levels were measured with a rat insulin enzyme-linked immune absorbent assay kit (ALPCO Diagnostics, Windham, NH, USA) according to the manufacturer’s protocol. GLP-1 (GLP-1 EIA Kit, Sigma-Aldrich, St. Louis, MO, USA), C-peptide (C-Peptide EIA Kit, Sigma-Aldrich, St. Louis, MO, USA), and HbA1c ELISA kit (Cusabio Biotech Co., Ltd., Wuhan, China) were quantitated using commercially available kits according to the manufacturers’ protocols.

### 2.7. Measurement of Antioxidant Defense

The serum concentrations of malondialdehyde (MDA) were measured with an OXI-TEK TBARS kit (Enzo Life Sciences Incorporated; Plymouth Meeting, PA, USA). The reaction products were determined by measuring the absorbance at 532 nm using a SpectraMax M2 microplate reader (Molecular Devices, Sunnyvale, CA, USA) according to the manufacturer’s instructions. The serum levels of superoxide dismutase (SOD) were quantitated using a SOD activity kit (Enzo Life Sciences Incorporated) measuring the absorbance of the reaction products at 450 nm. The levels of total glutathione (tGSH) and oxidized glutathione (GSSG) were measured using a glutathione (total) detection kit from Enzo Life Sciences Incorporated. The absorbance of the reaction products was read at 405 nm. As per the manufacturer’s protocol, the concentration of reduced glutathione (GSH) was calculated using the formula, GSH = (tGSH – GSSG). The redox ratio was calculated using the formula, GSH:GSSG = (tGSH − 2 GSSG)/GSSG), as previously described [18].

### 2.8. Histological Analysis

For histological analysis, the pancreases, livers, and kidneys were dissected from all of the study groups at the end of experiment period. The tissues were washed in normal saline, cut into pieces of the desired size, and fixed in 10% neutral buffered formalin solution. After fixation, the samples were cleaned and embedded in paraffin. Tissue sections of 5 μm thickness were mounted on slides, stained with hematoxylin-eosin (H-E), and examined on a light microscope.

### 2.9. Statistical Analysis

Data are expressed as means ± standard errors of the mean (SEMs). Differences between groups were evaluated by analysis of variance (ANOVA) with the Bonferroni *post hoc* test or by calculation of Spearman’s rank correlation coefficient, as appropriate, using Prism 5.03 (GraphPad Software Inc., San Diego, CA, USA). Statistical significance was set at *p* < 0.05.

## 3. Results

### 3.1. Effects of the Different Treatments on BW, Blood Glucose Levels, and Serum Insulin Levels

**Figure 1:**
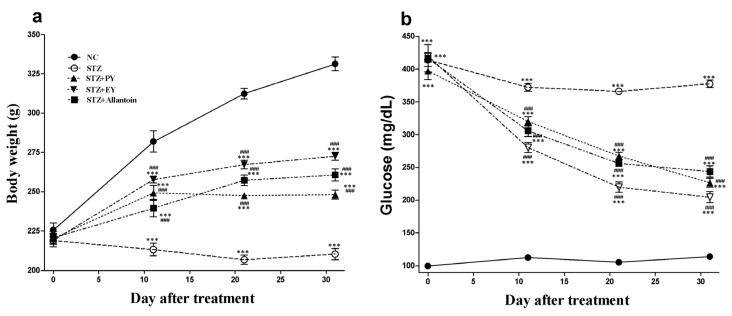
Effects of powdered yam (PY), extract of yam (EY), and allantoin on body weight (**a**) and fasting blood glucose (**b**) in streptozotocin (STZ)-induced diabetic rats. Data are reported as means ± SEMs (*n* = 10). *: *p* < 0.05 and ***: *p* < 0.001, Bonferroni *post hoc* test following one-way ANOVA *versus* the NC group (Cont); ^#^: *p* < 0.05; ^##^: *p* < 0.01; and ^###^: *p* < 0.001, Bonferroni *post hoc* test following two-way ANOVA *versus* the diabetic control group (STZ).

The effects of PY, EY, and allantoin on BW and FBG in STZ-induced diabetic rats are shown in Figure 1. During the study period, the normal control rats gained weight, while STZ-induced diabetic rats exhibited a lower body weight. The mean BWs of the treatment groups were significantly higher than those of the diabetic control group.

The mean FBG in diabetic rats was significantly increased compared with the normal control rats, whereas all the treatment groups showed significantly decreased blood glucose levels compared with the diabetic control group from Day 11 until the end of the experiment. Among all the groups, the EY-treated diabetic rats showed the lowest blood glucose levels.

### 3.2. Effects of the Different Treatments on Serum Insulin, GLP-1, C-Peptide, HbAlc, and Nitric Oxide Levels

**Figure 2:**
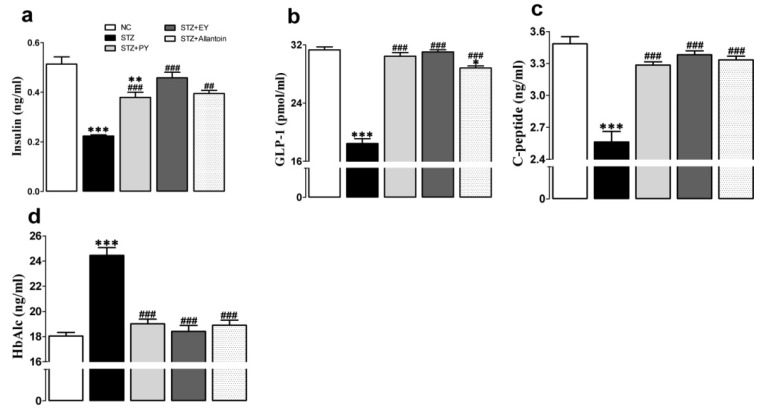
Effects of PY, EY, and allantoin treatments on the serum levels of insulin (a), GLP-1 (b), C-peptide (c), and HbAlc (d) in STZ-induced diabetic rats: GLP-1, glucagon-like peptide-1; HbA1c, glycated hemoglobin. Data are reported as means ± SEMs (*n* = 10). *: *p* < 0.05; **: *p* < 0.01; and ***: *p* < 0.001, Bonferroni *post hoc* test following one-way ANOVA *versus* the NC group (Cont); ^#^: *p* < 0.05; ^##^: *p* < 0.01; and ^###^: *p* < 0.001, Bonferroni *post hoc* test following one-way ANOVA *versus* the diabetic control group (STZ).

At the end of the experiment, the serum insulin, GLP-1, and C-peptide levels of diabetic control rats were significantly decreased compared to those of normal control rats. In contrast, the administration of PY, EY, or allantoin to diabetic rats significantly increased all of these parameters compared with the diabetic control rats. However, HbAlc significantly increased in diabetic rats, which were found near to normal in the treatment group (Figure 2).

### 3.3. Effects of the Different Treatments on the Serum Levels of Metabolic Enzymes, BUN, CRE, and UA

**Figure 3:**
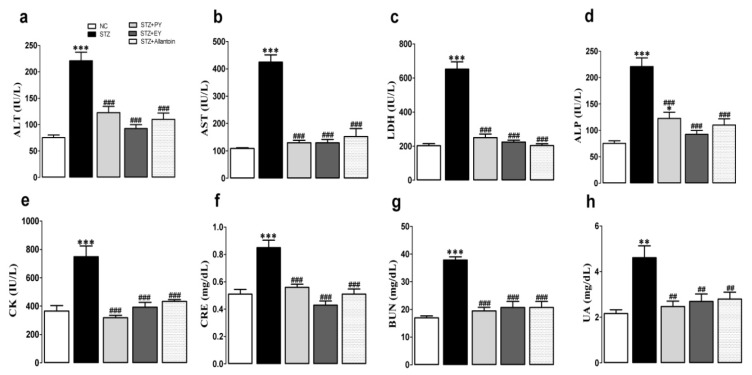
Effects of PY, EY, and allantoin treatments on the serum levels of metabolic enzymes, BUN, CRE, and UA in STZ-induced diabetic rats. ALT, alanine aminotransaminase (a); AST, aspartate aminotransferase (b); LDH, lactate dehydrogenase (c); ALP, alkaline phosphatase (d); CK, creatinine kinase (e); CRE, creatinine (f); BUN, blood urea nitrogen (g); UA, uric acid (h). Data are reported as means ± SEMs (*n* = 10). *: *p* < 0.05; **: *p* < 0.01; and ***: *p* < 0.001, Bonferroni *post hoc* test following one-way ANOVA *versus* the NC group (Cont); ^#^: *p* < 0.05; ^##^: *p* < 0.01; and ^###^: *p* < 0.001, Bonferroni *post hoc* test following one-way ANOVA *versus* the diabetic control group (STZ).

As shown in Figure 3, the serum levels of ALT, AST, ALP, LDH, CK, BUN, CRE, and UA were significantly increased in the diabetic control rats compared with the normal control rats. However, these elevations were significantly reduced in the yam-treated (PY and EY) and allantoin-treated groups compared with the diabetic control group.

### 3.4. Effects of the Different Treatments on Serum Lipid and Protein Levels

**Figure 4:**
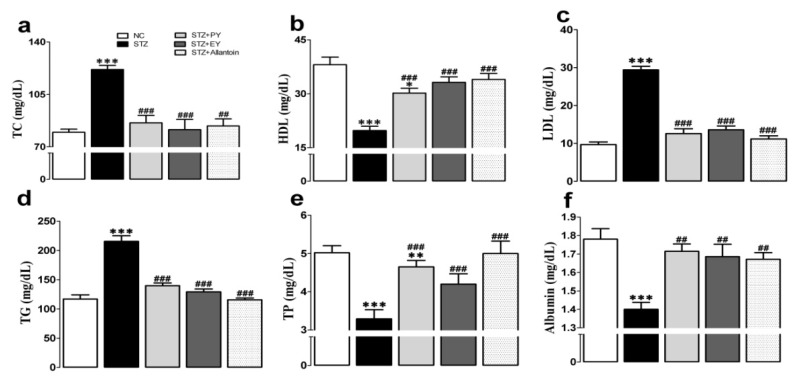
Effects of PY, EY, and allantoin treatments on serum lipid and protein profiles in STZ-induced diabetic rats. TC, total cholesterol (a); HDL, high density lipoprotein (b); LDL, low density lipoprotein (c); TG, triglyceride (d); TP, total protein (e); Albumin (f). Data are reported as means ± SEMs (*n* = 10). *: *p* < 0.05; **: *p* < 0.01; and ***: *p* < 0.001, Bonferroni *post hoc* test following one-way ANOVA *versus* the NC group (Cont); ^#^: *p* < 0.05; ^##^: *p* < 0.01; and ^###^: *p* < 0.001, Bonferroni *post hoc* test following one-way ANOVA *versus* the diabetic control group (STZ).

The serum levels of TC, LDL, and TG were markedly elevated in the diabetic group compared with the normal group, whereas the opposite effect was observed on the serum levels of LDL, TP, and albumin. However, these effects were reduced in the treatment groups (Figure 4).

### 3.5. Effects of the on Different Treatments Antioxidant Activities

**Figure 5:**
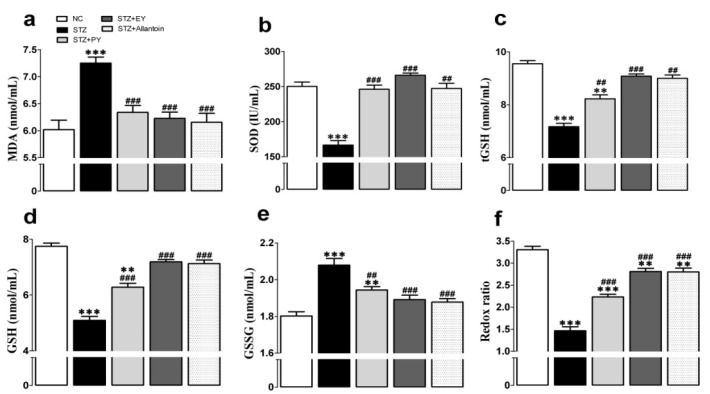
Effects of PY, EY, and allantoin treatments on serum lipid peroxidation and antioxidant activities in STZ-induced diabetic rats. MDA. Malondialdehyde (a); SOD, superoxide dismutase (b); tGSH, total glutathione (c); GSH, reduced glutathione (d); GSSG, oxidized glutathione (e); GSH/GSSG, ratio of GSH to GSSG (redox ratio) (f). Data are reported as means ± SEMs (*n* = 10). *: *p* < 0.05; **: *p* < 0.01; and ***: *p* < 0.001, Bonferroni *post hoc* test following one-way ANOVA *versus* the NC group (Cont); ^#^: *p* < 0.05; ^##^: *p* < 0.01; and ^###^: *p* < 0.001, Bonferroni *post hoc* test following one-way ANOVA *versus* the diabetic control group (STZ).

The serum concentrations of tGSH, GSH, and SOD were significantly decreased in the diabetic group compared with the normal group. These changes were accompanied by a significant decrease in the redox ratio (GSH:GSSG), an increase in the serum concentration of oxidized glutathione, and an increase in lipid peroxidation (MDA). However, these effects were lessened after 31 days of PY, EY, or allantoin administration (Figure 5).

### 3.6. Effects of the Different Treatments on Blood Ion and Metabolite Concentrations

As shown in Table 1, the blood pH, HCO_3_^−^ concentration, Hb concentration, and hematocrit values were markedly decreased, while the blood lactate concentration and osmolarity were increased in the diabetic group. Similarly, alterations in the concentrations of various ions (Mg^2+^, Ca^2+^, K^+^, and Cl^−^) were observed in this group. After treatment, most of these parameters had returned to normal.

**Table 1:** Effects of PY, EY, and allantoin on the blood hemoglobin concentration, hematocrit value, and electrolytic balance in STZ-induced diabetic rats.

	NC	STZ	STZ + PY	STZ + EY	STZ + Allatoin
Hb (g/dL)	13.4 ± 0.3	10.8 ± 0.3	13.2 ± 0.4 ^***^	13.5 ± 0.2 ^*^	13.3 ± 0.1 ^***^
Hct (%)	42 ± 1	36 ± 1 ^***^	41 ± 1 ^##^	42 ± 1 ^###^	41 ± 1 ^**^
pH	7.41 ± 0.01	7.33 ± 0.01 ^***^	7.43 ± 0.01 ^###^	7.43 ± 0.01 ^###^	7.44 ± 0.01 ^##^
Lactate (mmol/L)	3.9 ± 0.4	6.0 ± 0.3 ^**^	4.2 ± 0.2 ^###^	4.3 ± 0.3 ^##^	4.4 ± 0.4 ^#^
HCO_3_^−^ (mmol/L)	27.54 ± 0.58	23.63 ± 0.87 ^*^	26.86 ± 0.36	29.24 ± 0.40 ^##^	28.97 ± 0.069 ^##^
Osm (mOsm/L)	298 ± 1	324 ± 1 ^***^	307 ± 3 ^*,##^	298 ± 1 ^###^	302 ± 3 ^*,###^
Angap (mmol/L)	13.5 ± 0.7	14.7 ± 1.0	13.6 ± 0.8	12.8 ± 0.3	12.9 ± 1.1
Na^+^ (mmol/L)	149 ± 0	151 ± 0 ^*^	150 ± 1	148 ± 0 ^#^	150 ± 1
Cl^−^ (mmol/L)	108 ± 1	113 ± 1 ^*^	112 ± 2	106 ± 0 ^##^	110 ± 1
Mg^2+^ (mmol/L)	0.44 ± 0.11	0.41 ± 0.01	0.44 ± 0.01 ^###^	0.43 ± 0.01 ^###^	0.44 ± 0.02 ^###^
K^+^ (mmol/L)	4.3 ± 0.1	5.0 ± 0.2 ^*^	4.5 ± 0.2	4.3 ± 0.1 ^#^	4.3 ± 0.1 ^#^
Ca^2+^ (mmol/L)	1.11 ± 0.01	1.02 ± 0.02 ^*^	1.14 ± 0.03 ^#^	1.15 ± 0.02 ^##^	1.12 ± 0.01 ^#^

NC, normal control; STZ, diabetic control; STZ + PY, crude yam powder-treated diabetic; STZ + EY, water extract of yam-treated diabetic; STZ + Allantoin, allatoin-treated diabetic rats; Hb, hemoglobin; Hct, hematocrete; HCO_3_^−^, bicarbonate ion; Osm, osmolality; Angap, anionic gap. Data are reported as means ± SEMs (*n* = 10). *: *p* < 0.05; **: *p* < 0.01; and ***: *p* < 0.001, Bonferroni *post hoc* test following one-way ANOVA *versus* the NC group (Cont); ^#^: *p* < 0.05; ^##^: *p* < 0.01; and ^###^: *p* < 0.001, Bonferroni *post hoc* test following one-way ANOVA *versus* the diabetic control group (STZ).

### 3.7. Histological Analysis

**Figure 6:**
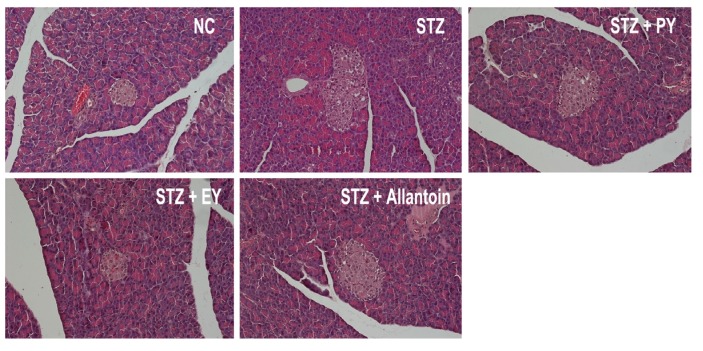
Microphotographs of pancreatic tissues (H-E staining, 200×). NC, normal control; STZ, diabetic control; STZ + PY, crude yam powder-treated diabetic; STZ + EY, water extract of yam-treated diabetic; STZ + Allantoin, allatoin-treated diabetic rats.

**Figure 7:**
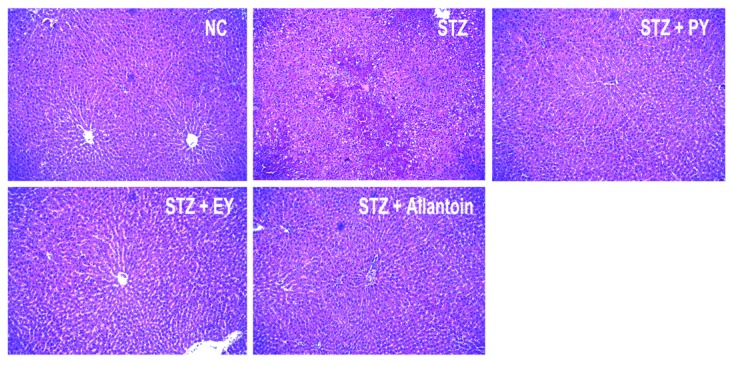
Microphotographs of hepatic tissues (H-E staining, 100×). NC, normal control; STZ, diabetic control; STZ + PY, crude yam powder-treated diabetic; STZ + EY, water extract of yam-treated diabetic; STZ + Allantoin, allatoin-treated diabetic rats.

**Figure 8:**
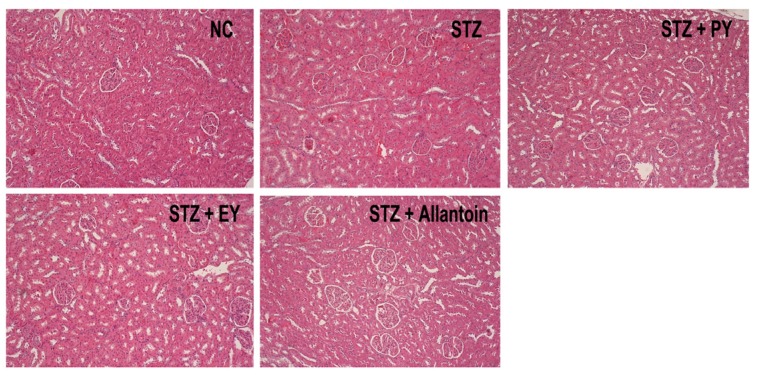
Microphotographs of renal tissues (H-E staining, 100×). NC, normal control; STZ, diabetic control; STZ + PY, crude yam powder-treated diabetic; STZ + EY, water extract of yam-treated diabetic; STZ + Allantoin, allatoin-treated diabetic rats.

In normal control rats, histological analysis revealed normal pancreatic beta-cells in the islet of Langerhans and the acini (Figure 6). Conversely, STZ-induced diabetic rats exhibited extensive granulation of beta cells and severe vacuolation of the pancreatic islets. Histological analysis of diabetic rats treated with PY, EY, and allantoin revealed comparatively less beta-cell granulation and reduced pancreatic islet vacuolation compared with diabetic control rats. Liver investigation of the control animals showed normal architecture of hepatic lobules in the form of hepatocytes arranged from the portal vein. Examination of rat livers from the diabetic group showed necrosis and loss of architecture of hepatocytes, slight hydropic degeneration, apoptotic nuclei, occasional binucleation, cellular infiltration, hemorrhage, and congestion of central veins Histopathological changes induced by STZ were remarkably improved by administration with yam and allantoin (Figure 7). Morphological changes were observed in the kidneys of the diabetic rats, including necrotic and damaged glomerili and renal tubular epithelial cells, vacuolization of the glomerular matrix but the lesions were absent in the NC group. However, yam and allantoin treated rats did not show any visible pathological changes (Figure 8).

## 4. Discussion

Our results show that yam exerts antidiabetic activity in streptozotocin-induced diabetic rats when administered either as a crude powder or as a water extract. Similar results were observed for allantoin, its active ingredient. We found that all of the treatments decreased the levels of fasting blood glucose and HbAlc; conversely, the treatments increased the levels of insulin, GLP-1, and C-peptide. However, EY exerted a stronger antidiabetic effect than allantoin. This may be due to additional constituents of yam, which include dioscorin, sapogenins, choline, l-arginine, polysaccharides, and proteins.

STZ is commonly used for experimental induction of type I diabetes mellitus, selectively causing pancreatic islet β-cell cytotoxicity [19]. Hyperglycemia in diabetic conditions is believed to cause oxidative stress and toxicity in pancreatic β-cells, leading to their dysfunction and the eventual development of insulin resistance in peripheral tissues. However, antioxidants, plant extracts, and supplemental treatments have all been found to prevent these effects, indicating that they could provide protection to β-cells [1,19,20]. Yam has been demonstrated to have antioxidant effects *in vitro* [11,16,21]. In the present study, the treatments increased the serum antioxidant activities of tGSH, GSH, and SOD; in contrast, the levels of MDA and GSSG were decreased by the treatments. These findings suggest that yam-based treatments may be able to ameliorate the effects of chronic oxidative stress on β-cells and other bodily organs. Since allantoin is the main component of yam, this or other antioxidants in yam may directly or indirectly preserve and regenerate β-cells. Histological analysis revealed that the pancreatic tissue from STZ-induced diabetic rats exhibited shrinkage, necrosis, and a damaged β-cell population; in contrast, the treated diabetic animals exhibited improved pancreatic β-cell morphology and function. This finding indicates that the decreased blood glucose levels and increased insulin secretion levels are due to the improved function of pancreatic β-cells. Another important protein is GLP-1, which enhances insulin biosynthesis and transcription, improves β-cell function and mass, and reduces apoptosis in β-cells. GLP-1 also reduces glucagon secretion, attenuates gastric emptying, and decreases weight gain [20]. This protein is secreted from the L cells of the gastrointestinal mucosa in response to a meal and is also associated with gut flora modulation [22,23]. Interestingly, serum GLP-1 levels were completely restored in the STZ + PY and STZ + PE groups, but not in the STZ + Allantoin group. This finding may be explained by the observation that yam supplements act as probiotics that enrich beneficial gut microbiota and suppress the growth of bacterial pathogens. Since yam supplements are a good source of carbon and energy, they may improve bacterial community diversity and modulate short-chain fatty acid production in the hindgut [24]. Moreover, diabetes has been demonstrated to be an inflammatory disease [25]. *Dioscorea batatas* has been shown to exert anti-inflammatory effects [26]. The anti-inflammatory effects of probiotics help in treating low-grade inflammation, which often accompanies diabetes. IL-6, a multifunctional pro-inflammatory cytokine, affects the secretion of GLP-1 by intestinal L cells. The levels of circulating GLP-1 have been found to correlate with the concentration of systemic IL-6 and also with the concentrations of other markers of inflammation, suggesting that the regulation of GLP-1 is inflammation-dependent [19]. Thus, it is noteworthy that treatment with yam extract promoted the release of GLP-1 and improved the function of β-cells, thereby maintaining insulin and glucose levels.

Fasting blood glucose and HbAlc levels are important indicators for patients with diabetes who have microvascular disease, coronary heart disease, ischemic stroke, and/or retinopathy. The HbAlc level has been found to be directly proportional to the blood glucose concentration [1,27]. As expected, the HbA1c levels were increased in the diabetic control groups in our study, which were lowered by the three treatment protocols. This outcome could be due to improved insulin secretion [1]. C-peptide is a protein that joins the α- and β-chains of insulin in the pro-insulin molecule. During insulin synthesis, this protein is cleaved from pro-insulin and secreted in equimolar concentration as insulin from the β-cells. Persistent C-peptide elevation is associated with lower hyperglycemia, which is in turn associated with reduced complications. These findings suggest that the relationship of endogenous secretion with complications could be secondary to the effects of secretion on glycemic control. Diabetic patients with C-peptide levels ≥ 0.2 pmol/mL have been shown to have low fasting glucose and HbAlc values. Specifically, for every pmol/mL increase in the baseline stimulated C-peptide level, a 1% reduction in the HbA1c level was observed among intensively treated patients with diabetes [28].

STZ induced elevated levels of TG, TC, and LDL, but decreased the level of HDL. Hypertriglyceridemia and hypercholesterolemia are major risk factors in diabetes with respect to the development of atherosclerosis and coronary heart disease, which are secondary complications of diabetes [1]. We found that after 31 days of treatment the levels of TG, TC, and LDL were reduced while the serum levels of HDL were improved. Thus, yam could potentially be used to reduce long-term cardiovascular complications in patients with diabetes. These results agree with previous reports on the antilipidemic and anticholesterolic properties of yam [12,26,29].

Also as expected, the serum CRE, BUN, and UA levels were significantly increased in diabetic rats as a consequence of renal failure, extracellular dehydration, and protein catabolism, respectively [1,17]. In contrast, the treated groups exhibited significantly reduced levels of these indicators, suggesting that allation, yam powder, and its aqueous extract can all improve renal function, prevent extracellular dehydration, and avoid protein catabolism. The histological evidence of kidney also proved the renoprotective effects of yam and allantoin in this study. The kidney is a main organ that regulates ion and electrolyte homeostasis. Altered concentrations of Mg^2+^, Ca^2^, K^+^, Na^+^, Cl^−^, anion gap, and electrolyte balance are common features in patients with diabetes due to deteriorated kidney function or increased urinary loss. These symptoms were improved by the treatments here describe, indicating that they may have renoprotective and/or antioxidative properties [16,29,30]. The elevated plasma levels of ALT, AST, ALP, LDH, and CK in our study indicate hepatotoxicity and oxidative stress. These elevations were prevented by the yam and allantoin treatments, which indicate that they also have antioxidative and hepatoprotective abilities like as other *Dioscorea.* spp. [16,26,29]. In this study, the improved hepatic histological appearance in the treated groups also supported the hepatoprotective effects of yam. Moreover, the levels of TP and albumin in treated diabetic rats were close to normal, suggesting that the treatments might be beneficial for kidney and liver function. Lowered blood pH, reduced HCO_3_^−^, and increased lactate levels were observed in the diabetic indicating acidic blood, which may be a sign of ketoacidosis [31]. As with other symptoms, all the treatments reduced these effects, indicating that they promote the proper utilization of glucose thereby ameliorating ketosis. The increased blood Osm in the diabetic group may result from the elevated blood glucose and BUN levels [17,32]. Alternatively, it may result from insulin deficiency [32]. Importantly, the blood Osm was reduced after treatment.

## 5. Conclusions

**Figure 9:**
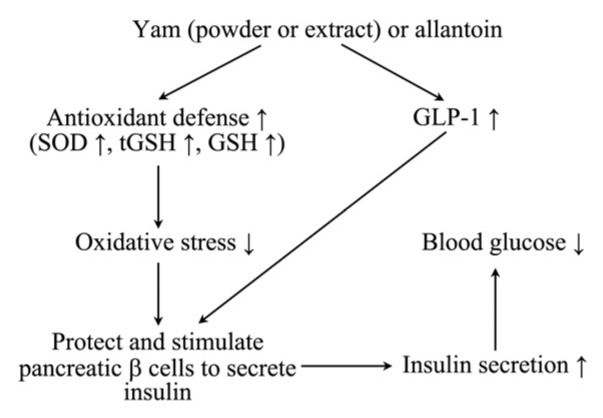
Schematic diagram of the proposed mechanisms by which powder or water extract of yam (*Dioscorea* *batatas*), and allantoin regulate blood glucose. Administration of yam or allantoin increased the GLP-1 level, responsible for enhancing insulin biosynthesis and transcription, improved β-cell function and mass, and reduced apoptosis in β-cells, controlling insulin and glucose levels. They also increased the antioxidant level and decreased the MDA level, which ultimately decreased oxidative stress protecting pancreatic β-cells and other organs.

The results of this study indicate that yam and allantoin exert antidiabetic effects. These two substances may modulate oxidative stress, antioxidant activities, and lipid profiles; improve kidney and liver function; promote the release of GLP-1; and improve the function of β-cells, thereby maintaining insulin and glucose levels (Figure 9).
